# Supplementary material for: The transcription factor OsSUF4 interacts with SDG725 in promoting H3K36me3 establishment
Source: Nat Commun. 2019 Jul 5;10:2999. doi: 10.1038/s41467-019-10850-5 (PMC6611904; doi:10.1038/s41467-019-10850-5)
Supplement: Supplementary file 1 — Supplementary Information [file 41467_2019_10850_MOESM1_ESM.pdf]

Supporting information

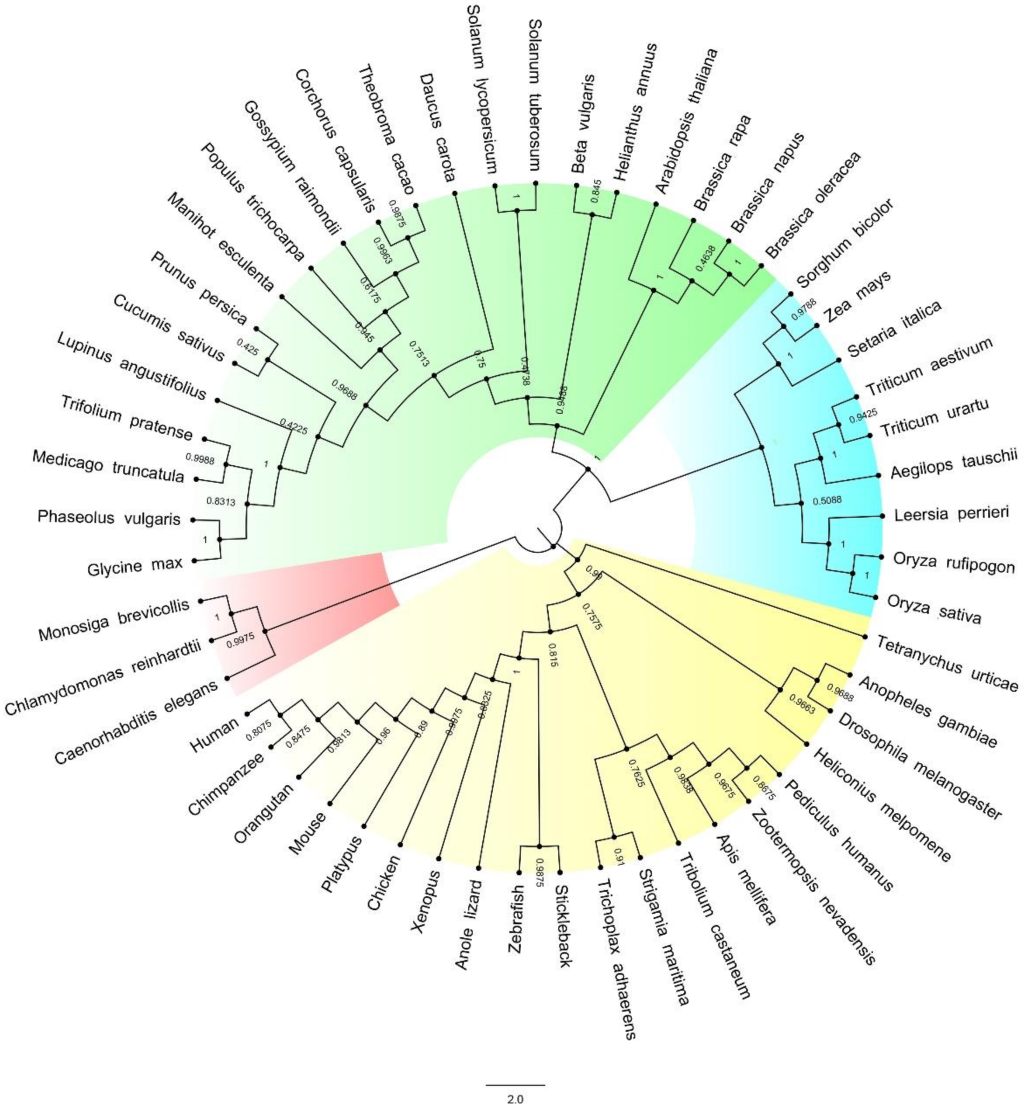

Supplementary Figure 1. OsSUF4 homologs are widespread in eukaryotes.

Phylogenic analysis of SUF4 proteins in eukaryotes using full-length amino acid sequences. Sectors with different colors represent different sub-clades.

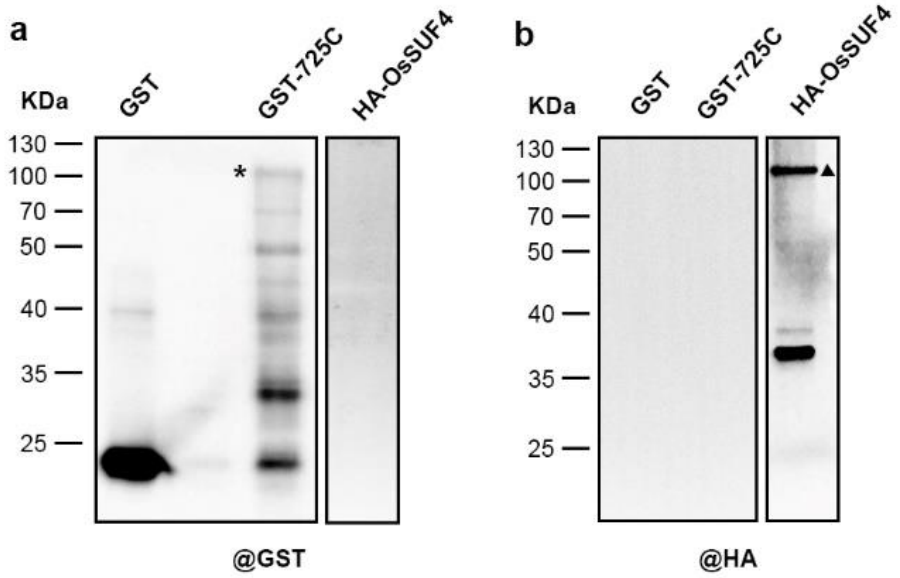

Supplementary Figure 2. GST, GST-SDG725C (GST-725C) and HA-OsSUF4 proteins detected by western blotting using antibody against GST or HA.

(**a**) The antibody against GST recognized GST and GST-725C proteins, but not HA-OsSUF4. GST-725C is marked with an asterisk. (**b**) The HA antibody recognized HA-OsSUF4 but not GST or GST-725C proteins. HA-OsSUF4 is marked with a triangle. Source data are provided as a Source Data file.

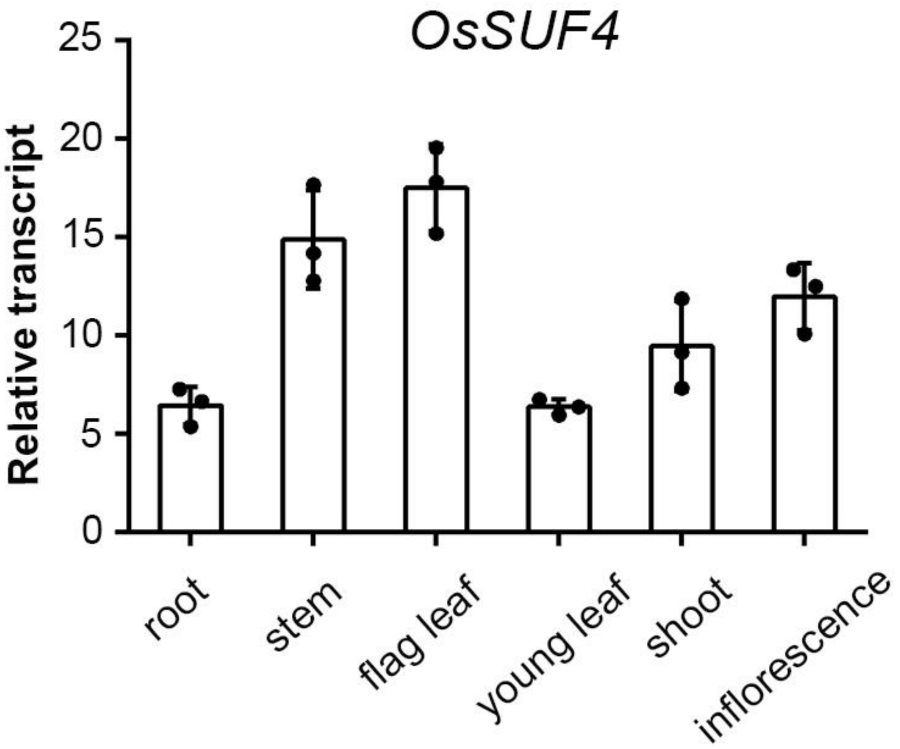

Supplementary Figure 3. *OsSUF4* is widely expressed in different tissues of rice. Relative transcription levels of *OsSUF4* were determined by qRT-PCR in different tissues, and normalized to the internal control *OsUbiquitin5*. Error bars representing mean ± SD are based on the average of three biological repeats. Source data are provided as a Source Data file.

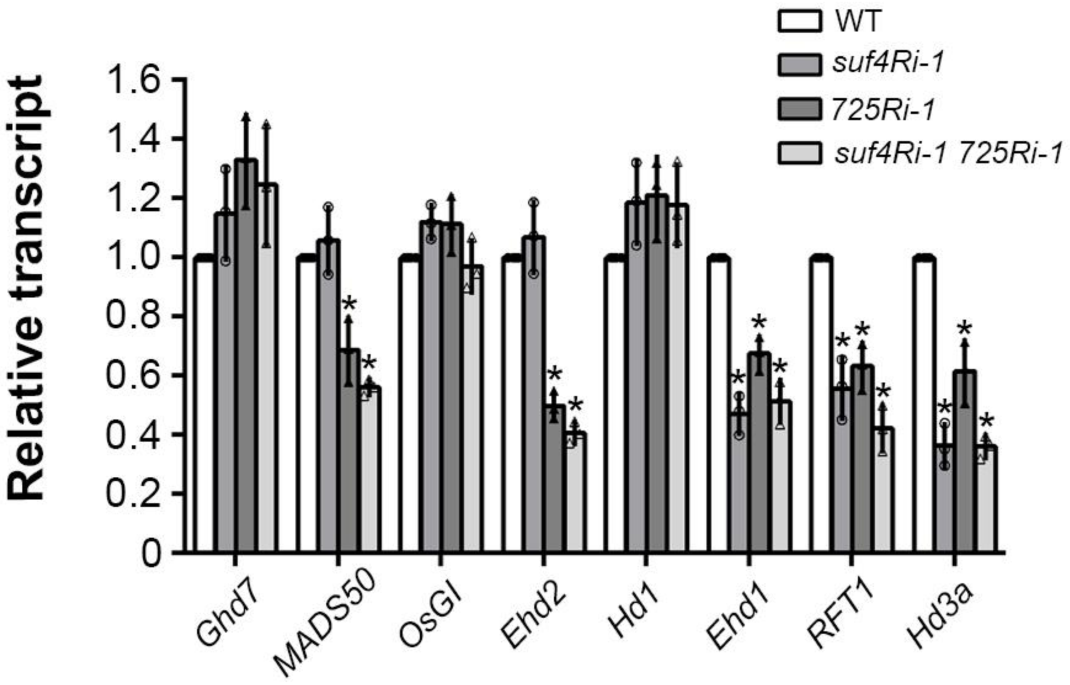

Supplementary Figure 4. Relative transcription levels of flowering genes in indicated genotypes under LD conditions.

35-day-old plants were collected 4 h from the start of illumination. Error bars representing mean ± SD are based on the average of three biological repeats normalized to the internal control *OsUbiquitin5*; and each value in WT was set as 1. Asterisks indicate significant differences between WT and mutants (Student's *t*-test: $*P < 0.01$). Source data are provided as a Source Data file.

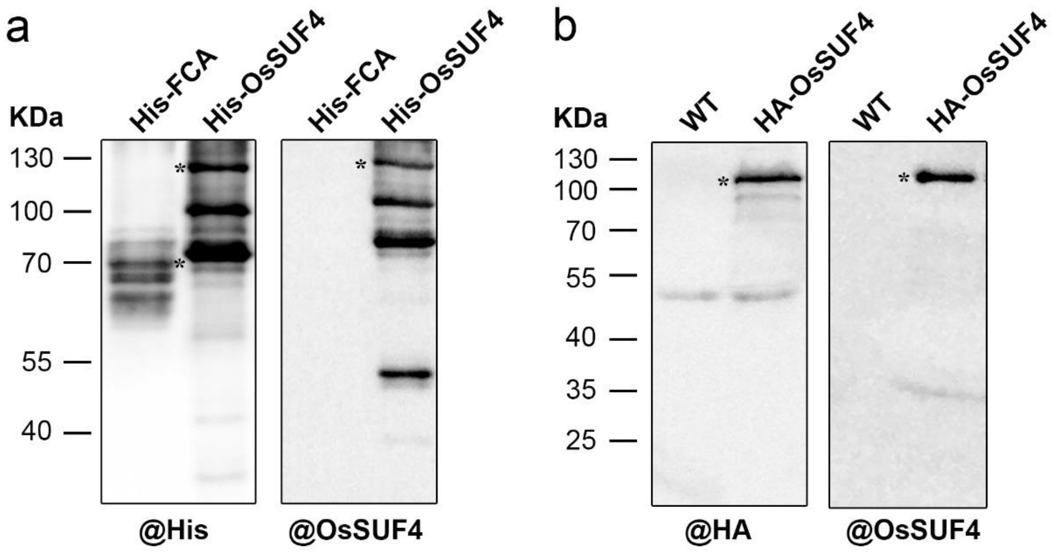

Supplementary Figure 5. Specificity of the antibody against OsSUF4.

Purified recombinant His-OsSUF4 from *E. coli* (**a**) and nuclear extracts from rice plants overexpressing *HA-OsSUF4* (**b)** were used to confirm the specificity of the monoclonal antibody against OsSUF4. His-FCA and WT served as negative controls. Immunized bands corresponding to full-length proteins are marked with asterisks. Source data are provided as a Source Data file.

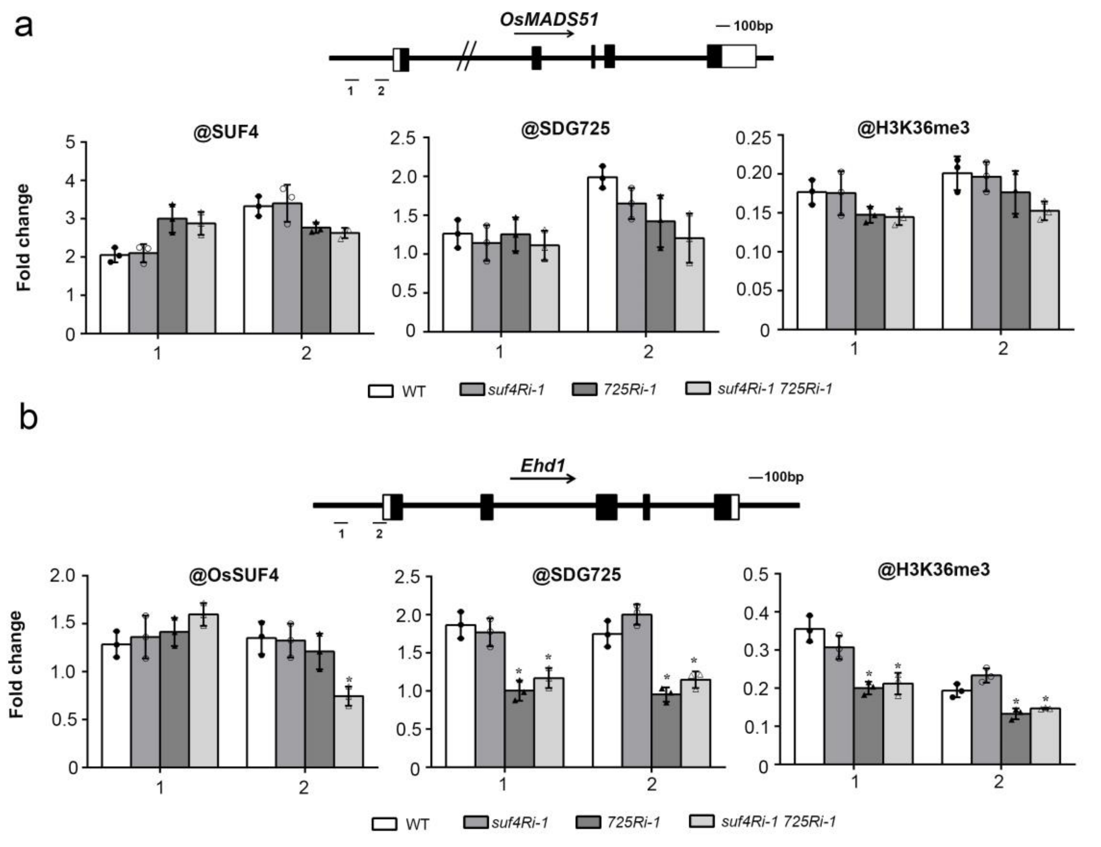

Supplementary Figure 6. *OsMADS51* and *Ehd1* are not target genes of OsSUF4.

ChIP analyses were performed at *OsMADS51* (**a**) and *Ehd1* (**b**) chromatin regions using antibodies against OsSUF4, SDG725, and H3K36me3 in WT, *suf4Ri-1*, *725Ri-1*, and *suf4Ri-1 725Ri-1* plants. Upper panels show the schematic representation of *OsMADS51* (**a**) and *Ehd1* (**b**) genes and the regions examined in ChIP-PCR experiments. Values are the mean ± SD of three individual biological replicates normalized to the internal control *OsUbiquitin5* (Student's *t*-test: $*P < 0.01$). Source data are provided as a Source Data file.

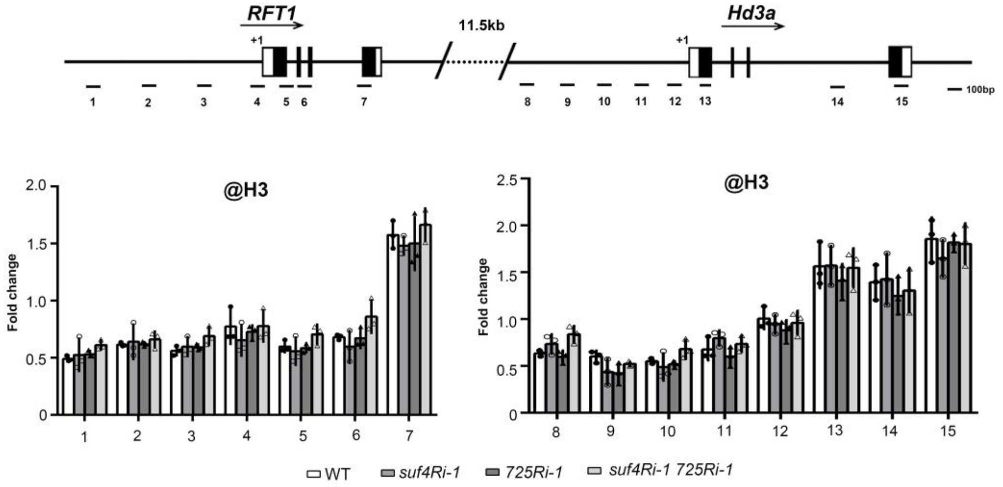

Supplementary Figure 7. ChIP analysis using antibody against H3 at *RFT1* and *Hd3a* chromatin in the indicated genotypes.

Upper panels show schematic representation of *RFT1* and *Hd3a* genes and the regions examined in ChIP-PCR experiments. Values are the mean ± SD of three individual biological replicates normalized to the internal control *OsUbiquitin5* (Student's *t*-test: **P* < 0.01). Source data are provided as a Source Data file.

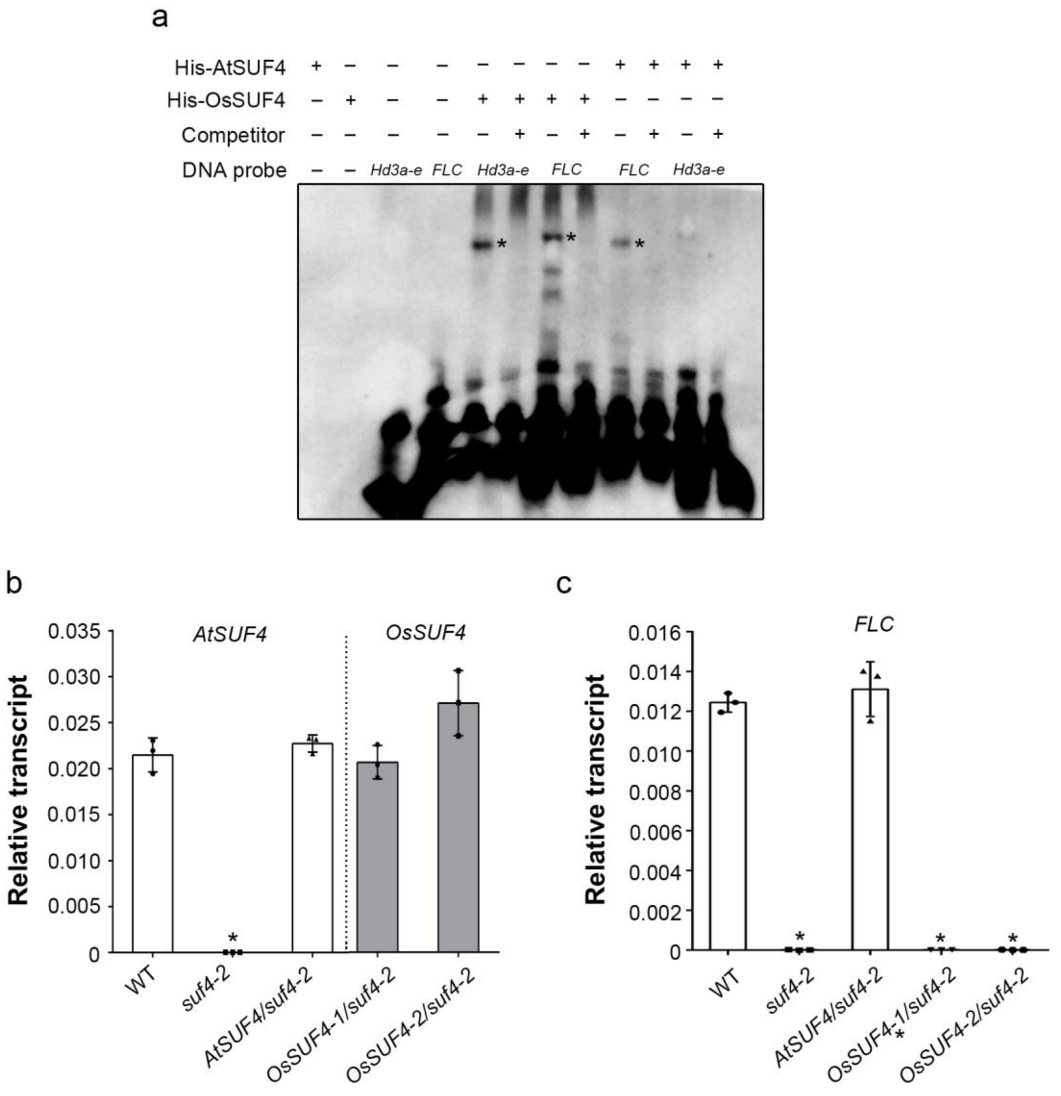

Supplementary Figure 8. *OsSUF4* failed to rescue Arabidopsis *suf4-2* mutant.

a, EMSA results show that OsSUF4 binds to the element within *FLC* promoter but AtSUF4 does not bind to that of *Hd3a*. Asterisks indicate the shifted bands. His-AtSUF4 and His-OsSUF4: the full-length purified recombinant proteins from *E. coli*. DNA probes: *Hd3a-e* (32-bp) and *FLC* (28-bp). Competitor: unlabeled DNA probes (100-fold excess). **b**, Relative transcription levels of *AtSUF* or *OsSUF4* were determined by qRT-PCR in Col (WT), *suf4-2*, *AtSUF4*/*suf4-2* and *OsSUF4*/*suf4-2* plants. Values shown are the mean ± SD of three independent biological replicates

normalized to the internal control gene *AtACT2* and the value of WT was set as 1 (Student's *t*-test: **P* < 0.01). **c**, Relative transcription levels of *FLC* were determined by qRT-PCR in WT, *suf4-2*, *AtSUF4*/*suf4-2* and *OsSUF4*/*suf4-2* plants. Values shown are the mean ± SD of three independent biological replicates normalized to the internal control gene *AtACT2* and the value of WT was set as 1 (Student's *t*-test: **P* < 0.01). Source data are provided as a Source Data file.

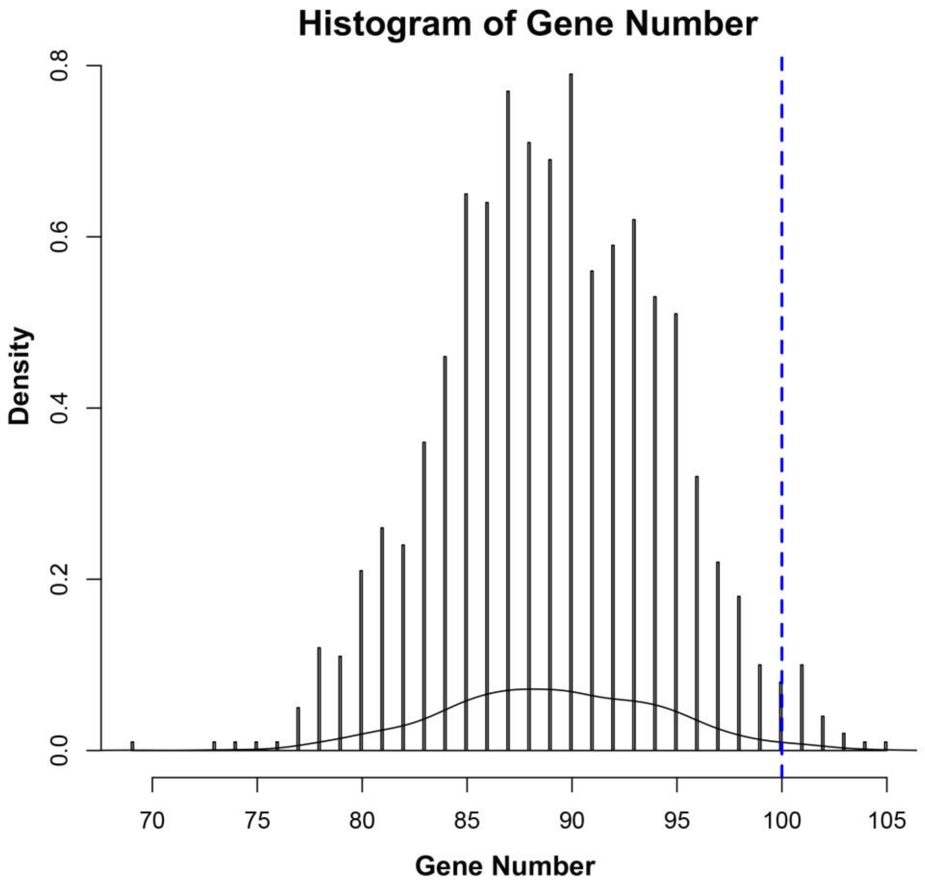

Supplementary Figure 9. The numbers of H3K36me3 enriched genes from 1000 sets.

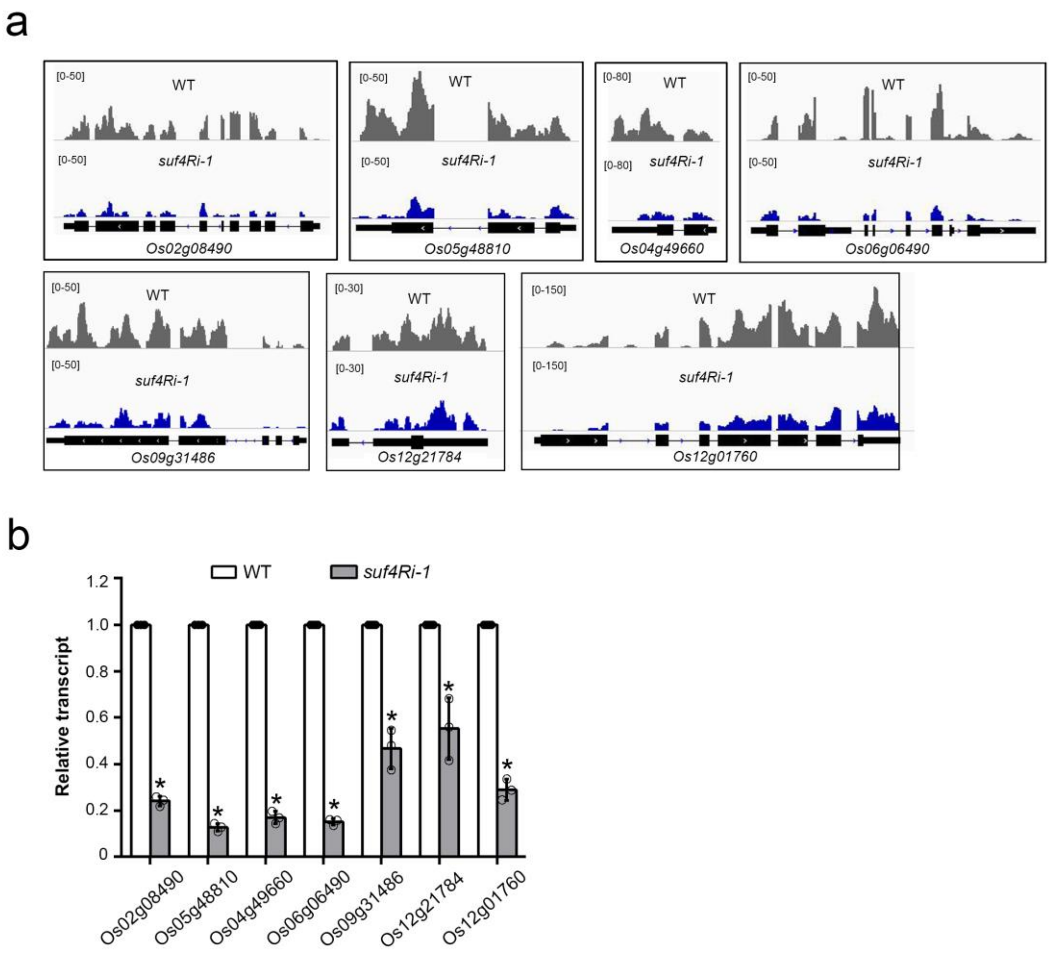

Supplementary Figure 10. Seven example genes containing the 7-bp element within the promoter region were significantly down-regulated in the *OsSUF4*-knockdown mutant.

a, IGV shots of RNA-seq showing the transcription levels of randomly selected genes containing the 7-bp DNA element in WT and *suf4Ri-1* plants. **b**, Seven genes down-regulated in the *suf4Ri-1* mutant were verified by qRT-PCR. Values are the mean ± SD of three independent biological replicates normalized to the internal control *OsUbiquitin5*; each value in WT was set as 1 (Student's *t*-test: *$P < 0.01$). Source data are provided as a Source Data file.

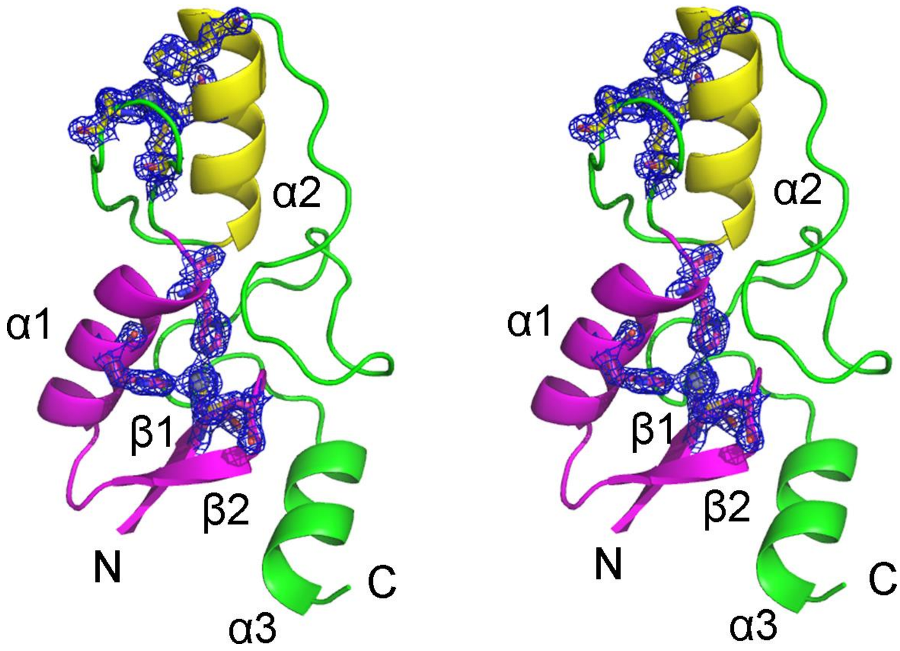

Supplementary Figure 11. A stereo view showing the overall structure of the zinc-finger domains of OsSUF4. The $2f_o$-f_c electron density map was contoured at 1.5 sigma level.

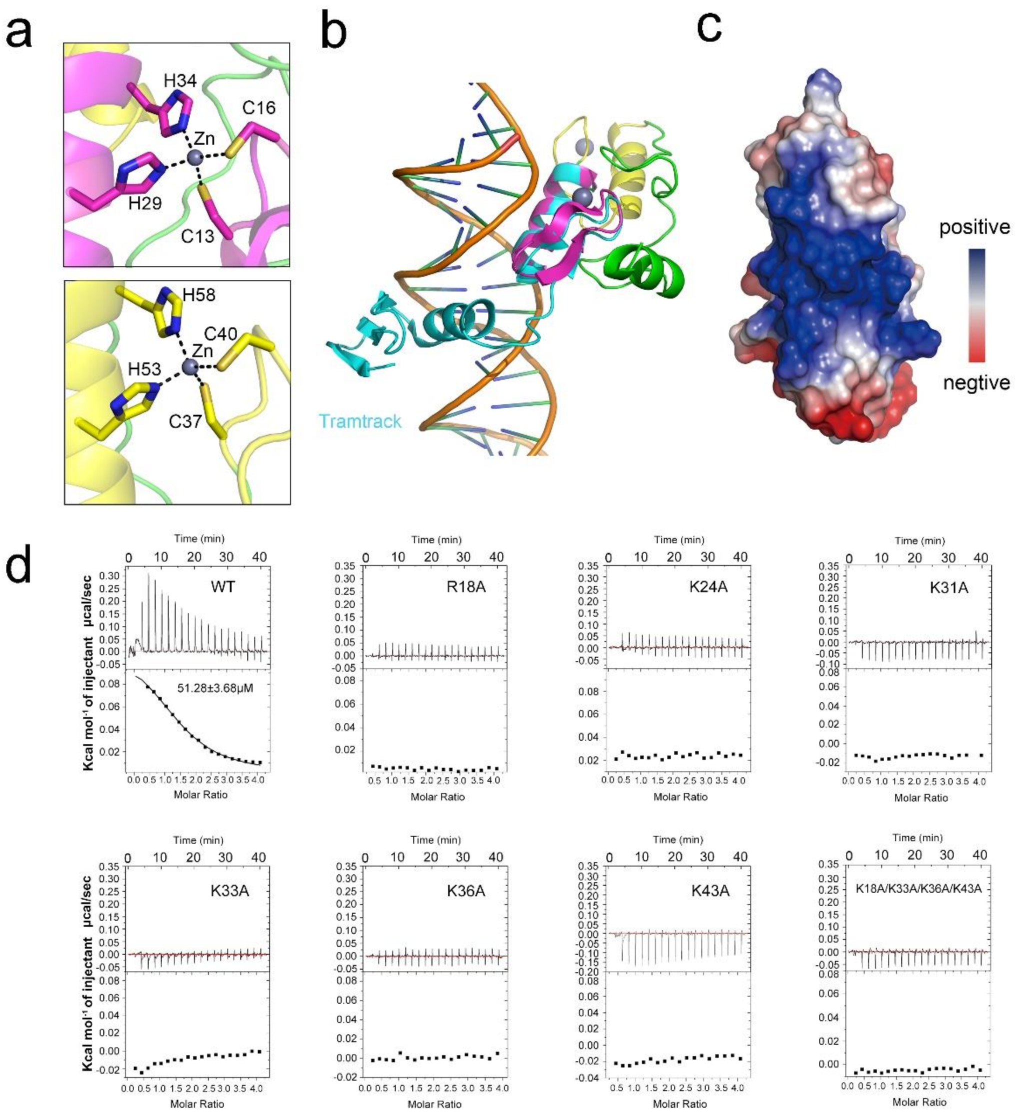

Supplementary Figure 12. The key residues of OsSUF4 involved in binding to target DNA.

a, The detailed coordination of the two zinc ions observed in the OsSUF4 structure. The zinc ions were shown as grey spheres. The residues involved in Zn-coordination were shown as sticks. **b,** Superposition of the zinc-finger domains of OsSUF4 and Tramtrack. The OsSUF4 structure was colored as in a. The zinc-finger domains and the DNA backbones were colored in cyan and orange in the Tramtrack structure, respectively. **c,** Electrostatic potential distribution of OsSUF4. **d,** ITC experiments

showing the binding between the target DNA (5’-GGGTACGGAAATGGTA-3’) and the wild-type or mutated OsSUF4.

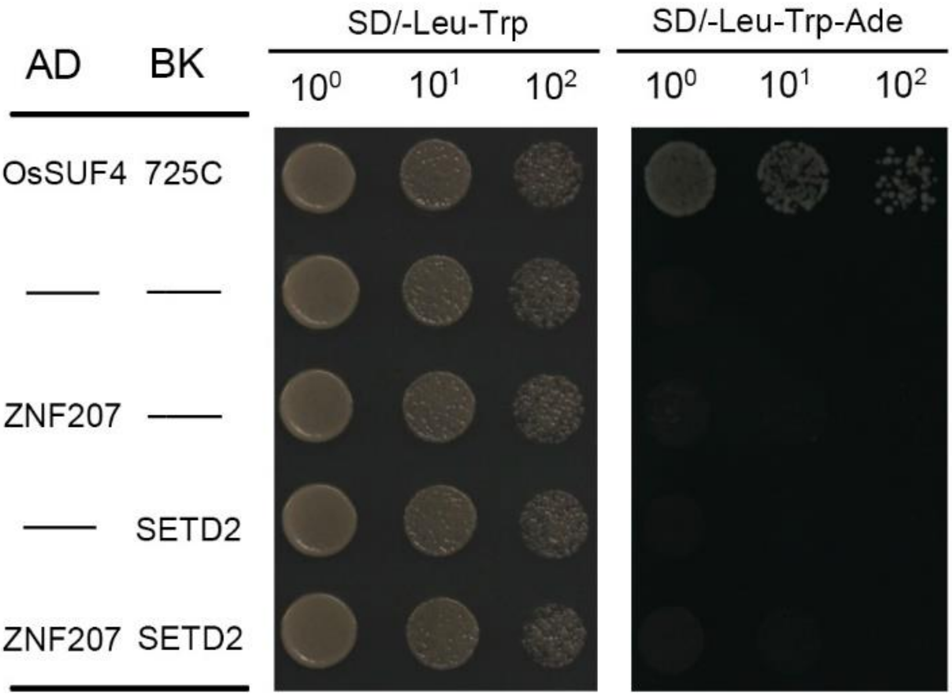

Supplementary Figure 13. Human ZNF207 failed to interact with SETD2 in yeast two-hybrid assay. SDG725 and OsSUF4 served as the positive control.

Supplementary Table 1 Data collection and refinement statistics

	SeMet OsSUF4(10-100)
PDB code	6J0D
**Data collection**	
Space group	$P4_12_12$
Cell dimensions	
a, b, c (Å)	74.0, 74.0 ,37.6
α, β, γ (°)	90.0, 90.0, 90.0
Wavelength (Å)	0.9796
Resolution (Å)[a]	52.34-1.90 (1.97-1.90)
R_{sym}	0.073(0.888)
$I / \sigma I$	24.7(3.6)
Completeness (%)	99.9(100)
Redundancy	10.3(11)
**Refinement**	
Resolution (Å)	52.34-1.90
No. reflections	8190
R_{work} / R_{free}	19.7/23.0
No. atoms	
Protein	691
Zinc ion	2
Water	37
Mean B-factors ($Å^2$)	24.3
R.m.s. deviations	
Bond lengths (Å)	0.007
Bond angles (°)	1.250
Ramachandran plot	
Most preferred (%)	95.3
Allowed (%)	4.7

[a] Values in parentheses are for the highest resolution shell.
