# Supplementary material for: The transcription factor OsSUF4 interacts with SDG725 in promoting H3K36me3 establishment
Source: Nat Commun. 2019 Jul 5;10:2999. doi: 10.1038/s41467-019-10850-5 (PMC6611904; doi:10.1038/s41467-019-10850-5)
Supplement: Supplementary file 3 — Description of Additional Supplementary Files [file 41467_2019_10850_MOESM3_ESM.pdf]

Description of Additional Supplementary Files

File Name: Supplementary Data 1
Description: Primers used in this study

File Name: Supplementary Data 2
Description: Genes containing the 7-bp element (5'-CGGAAAT-3') within the promoter region of the rice genome

File Name: Supplementary Data 3
Description: Genes down-regulated to 1.5-fold in the suf4Ri-1 mutant compared with WT.

File Name: Supplementary Data 4
Description: Genes enriched with H3K36me3 modification in WT plants
